# Inhibition of spleen tyrosine kinase decreases donor specific antibody levels in a rat model of sensitization

**DOI:** 10.1038/s41598-022-06413-2

**Published:** 2022-02-28

**Authors:** Shenzhen Tempest-Roe, Maria Prendecki, Stephen P. McAdoo, Candice Clarke, Anisha Tanna, Tabitha Turner-Stokes, Esteban S. Masuda, Michelle Willicombe, H. Terence Cook, Candice Roufosse, David Taube, Charles D. Pusey, Frederick W. K. Tam

**Affiliations:** 1 Department of Immunology & Inflammation, Centre for Inflammatory Disease, Imperial College London, London, UK; 2 Imperial College Healthcare NHS Trust, London, UK; 3 Rigel Pharmaceuticals Incorporated, South San Francisco, CA USA

**Keywords:** Allotransplantation, Humoral immunity

## Abstract

Antibody mediated rejection is a major cause of renal allograft loss. Circulating preformed donor specific antibodies (DSA) can result as a consequence of blood transfusion, pregnancy or prior transplantation. Current treatment strategies are limited due to partial or transient efficacy, adverse side-effects or patient unsuitability. Previous in vivo studies exploring autoimmune diseases have shown that spleen tyrosine kinase (SYK) signalling is involved in the development of pathogenic autoantibody. The role of SYK in allogenic antibody production is unknown, and we investigated this in a rodent model of sensitization, established by the transfusion of F344 whole blood into LEW rats. Two-week treatment of sensitized rats with selective SYK inhibitor fostamatinib strongly blocked circulating DSA production without affecting overall total immunoglobulin levels, and inhibition was sustained up to 5 weeks post-completion of the treatment regimen. Fostamatinib treatment did not affect mature B cell subset or plasma cell levels, which remained similar between non-treated controls, vehicle treated and fostamatinib treated animals. Our data indicate fostamatinib may provide an alternative therapeutic option for patients who are at risk of sensitization following blood transfusion while awaiting renal transplant.

## Introduction

Antibody mediated rejection (AMR) represents a significant barrier to allograft survival. The presence of preformed alloreactive donor specific antibodies (DSA) can arise from exposure to cells originating from other individuals via pregnancy, prior transplantation and blood transfusion^1^.

There is abundant research implicating the pathogenic role of DSA directed against polymorphic human leukocyte antigen class I (HLA I) and HLA class II (HLA II) in multiple organs. Non-HLA targets also play a crucial role in allograft rejection and DSA targets include minor histocompatibility molecules such as major histocompatibility complex (MHC) class I chain-related protein A (MICA) and major histocompatibility complex (MHC) class I chain-related protein B (MICB)^2^. Other commonly reported non-HLA targets are expressed on epithelial cells and endothelial cells^3, 4^.

There are two main mechanisms of allograft damage by DSA. The classical complement pathway can be activated by DSA binding to target antigen on endothelium, with generation of complement components which recruit inflammatory cells to the graft or directly damage the graft through the formation of the membrane attack complex^5^. In the process of antibody-dependent cell mediated cytotoxicity (ADCC), effector cells bearing Fcγ receptors (FcγR) can interact with the crystalline fragment (Fc) of bound DSA on endothelium and trigger lysis of target cells^6^.

Over 40% of patients awaiting kidney transplant in the United Kingdom are presensitized, with a reported median wait time of 6.1 years, which is double that of non-sensitized patients^7^. Current desensitization strategies include plasmapheresis and intravenous immunoglobulin, with or without immunosuppression using the B cell depletion-agent rituximab^8, 9^. However, these strategies are limited and have partial or transient efficacy, in addition to their adverse effects and unsuitability for patients with certain comorbidities^10^. It is crucial therefore to identify new, safer and more effective therapeutic targets for desensitization of patients.

Spleen tyrosine kinase (SYK) is a cytosolic non-receptor tyrosine kinase mainly expressed in haemopoietic cells. Activation of SYK and subsequent signal transduction is initiated downstream of classical immunoreceptors including FcγR and the B cell receptor (BCR). In the B cell, SYK signalling plays a critical role in B cell maturation and effector functions^11–14^. Increasing numbers of studies have targeted SYK for the treatment of immune and inflammatory diseases, and it has potential efficacy in the treatment of presensitized patients^15–18^. Fostamatinib is a small molecule SYK inhibitor, and through its active metabolite R406 has previously shown efficacy in the treatment of experimental autoimmune glomerulonephritis in rats, where treatment resulted in attenuation of autoantibody production^19^.

Here, in a rat model of sensitization, we demonstrate that fostamatinib treatment is able to prevent the production of allogenic antibody, even after cessation of treatment, maintaining overall non-allogenic immunoglobulin levels, whilst having no depletory effect on the levels of plasma cell or B cell populations.

## Materials and methods

### Animals and transfusions

Eight-week old LEW.Crl RT1^I^ (LEW) and F344/DUCrl RT1^lv^ (F344) male rats were purchased from Charles River UK Ltd (Margate, UK) and maintained in a pathogen-free animal facility at the Central Biomedical Services unit, Hammersmith Hospital Campus, Imperial College London. LEW received 800 µL heparinised whole blood from F344 via an intravenous route. All animal studies were licensed by the Home Office Science Unit. Studies and procedures were approved by Imperial College London Research Ethics committee and carried out in accordance with the regulations of the UK Animals (Scientific Procedures) Act (1986) and ARRIVE (Animal Research: Reporting of In Vivo Experiments) guidelines.

### Treatments

SYK specific inhibitor fostamatinib was provided by Rigel Pharmaceuticals (South San Francisco, CA, USA). Fostamatinib or vehicle (0.1% carboxymethylcellulose) was administered in sensitized animals by oral gavage twice daily for a period of 14 days at 40 mg/kg from either 24 h or 7 days post-transfusion.

### Detection of total serum immunoglobulin

Serum levels of total IgG and IgM were detected with Enzyme Linked Immunosorbent Assay (ELISA) from ThermoFischerScientific. Serum was diluted by 1:10,000 (IgM) or 1:50,000 (IgG).

### Flow cytometry

Samples were analysed on a BD LSR Fortessa™ X-20 and data analysed on FlowJo 9.6.6 software.

#### Flow crossmatch analysis

IgG and IgM alloantibodies were detected by flow cytometry. 1 × 10^6^ F344l splenocytes were suspended in RPMI-1640 medium containing 10% FCS and incubated with diluted recipient LEW (1/50) sera at 37 °C for 30 min. Splenocytes were incubated with the following anti-rat antibodies for 20 min in 0.5% BSA: anti-IgM (MRM-47 Biolegend), anti-IgG (Poly4054 Biolegend), anti-IgG1 (MARG1-2 AbCam), anti-IgG2a (MRG2A-83 Biolegend), anti-IgG2b (MRG2b-85 Biolegend), IgG2c (R2C-23A3 eBioscience), anti-CD3 (14F Biolegend) and anti-CD45 (OX-1 Biolegend). Alloreactivity was analysed by determining the mean fluorescence intensity (MFI) on gated live CD45^+^ (OX-1 Biolegend) CD3^+^ (14F Biolegend) cells.

#### B cell and plasma cell subset analysis

Splenocytes were isolated from LEW rats and incubated with the following antibodies: anti-CD3 (1F4 Biolegend), anti-CD45 (OX1 Biolegend), anti-CD45R (HIS24 BDBiosciences), anti-CD27 (LG.3A10 Biolegend), anti-CD138 (DL-101 Santa Cruz Biotechnology), anti-IgD (MARD1 ThermoFisher Scientific), anti-IgM (MRM-47 Biolegend), anti-CD3 (14F Biolegend), and anti-CD45RA (OX-33 Biolegend).

Cells were gated to select singlets then CD3^-^ cells. Following this, plasma cells were selected by gating on IgD^−^CD45R^−^IgM^−^CD138^+^, memory cells by CD45R^+^CD27^+^, switched cells by CD45R^+^CD27^+^IgD^−^ and non-switched cells by CD45R^+^CD27^+^IgD^+^. Precision count beads (Biolegend) were used to obtain absolute cell counts.

### Statistical analyses

Data were analysed with GraphPad Prism 8.0 (GraphPad Software, Inc, La Jolla, CA) and are displayed as the mean ± SEM using a Mann–Whitney U test as appropriate. A P < 0.05 was defined as significant.

## Results

### Transfusion results in the production of donor specific antibody

**Figure 1:**
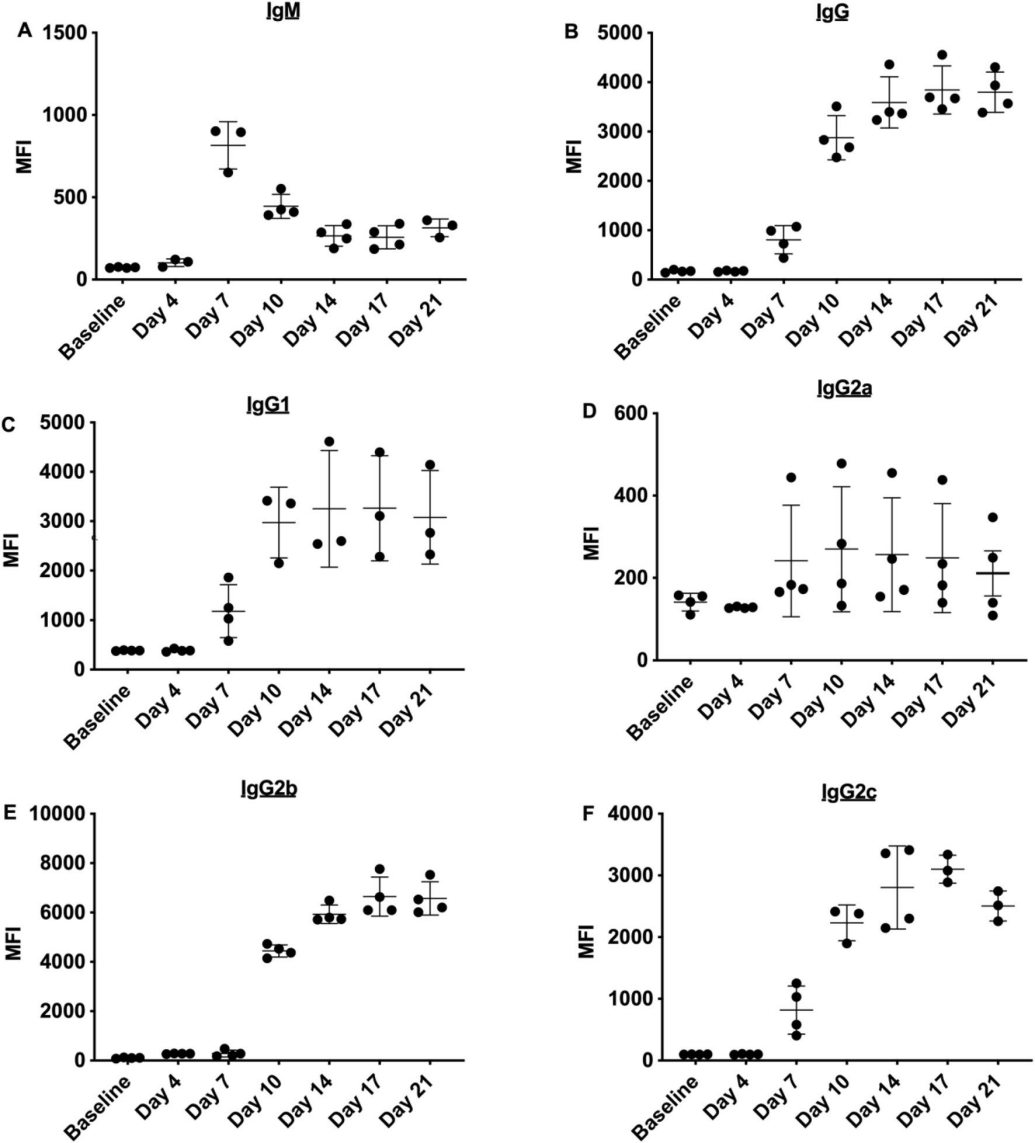
Whole F344 blood transfusion to LEW rats induces an alloantibody response. Flow cytometry T lymphocyte crossmatch was performed to detect allogenic (**A**) IgM levels, (**B**) IgG levels and IgG subsets (**C**) in sensitized LEW rats (n = 4).

A rat model of sensitization was established by whole blood transfusion between F344 and LEW rats. T lymphocyte (CD3^+^) flow crossmatch analysis showed that transfusion between these strains elicited an alloantibody response. An initial peak in allogenic IgM levels 7 days post-transfusion (Fig. 1A), was followed by isotype switching to IgG (Fig. 1B) where levels peaked at day 17. Additionally, all DSA IgG isotypes IgG1 (Fig. 1C), IgG2a (Fig. 1D), IgG2b (Fig. 1E), IgG2c (Fig. 1F) increased following transfusion.

### Treatment with fostamatinib inhibits allogenic antibody production

**Figure 2:**
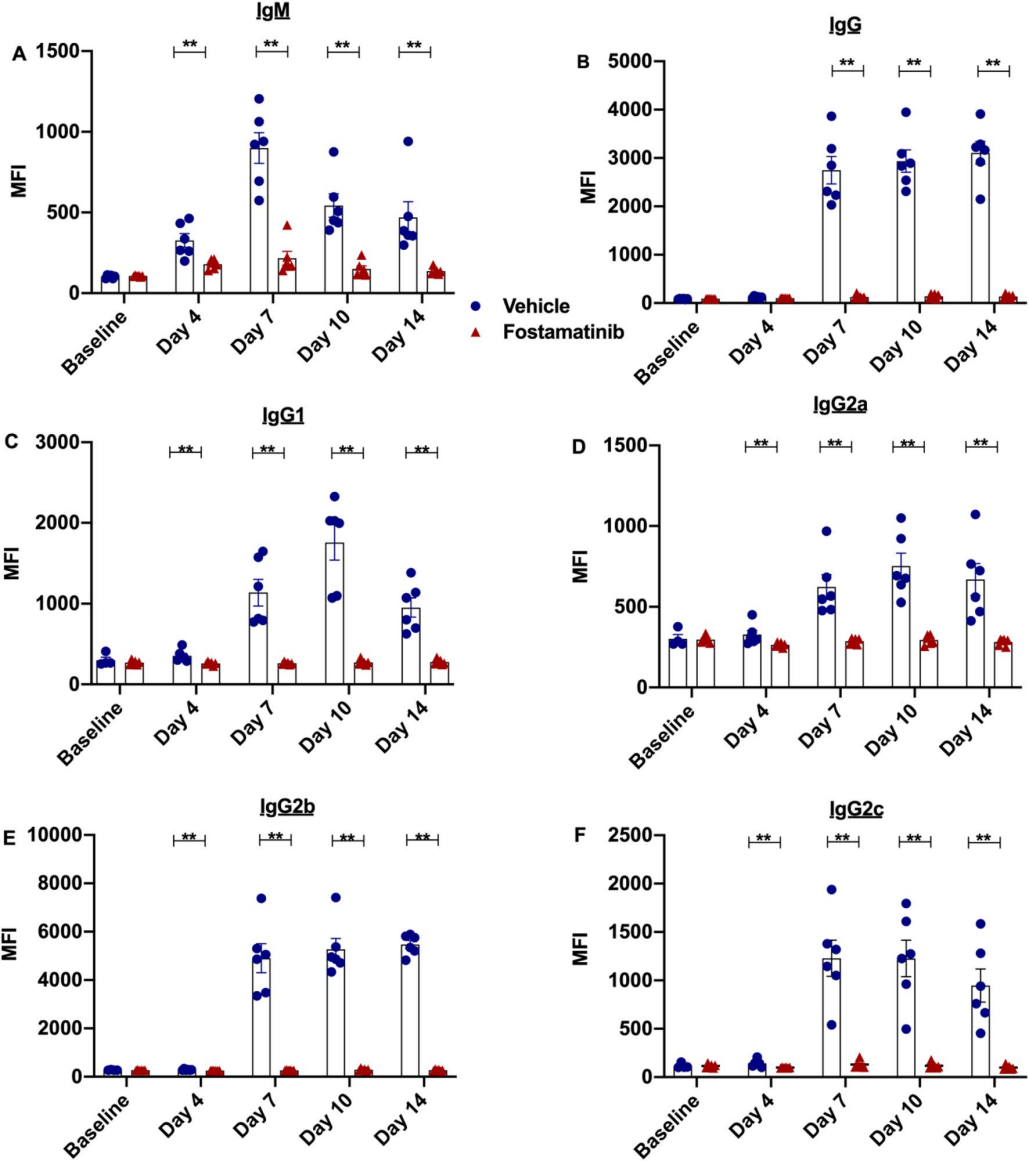
Fostamatinib treatment prevented production of donor specific antibody. T Lymphocyte crossmatch was performed to detect DSA levels in sensitized fostamatinib treated and vehicle treated LEW rats. (**A**) IgM, (**B**) IgG and (**C–F**) IgG subset production was inhibited by fostamatinib treatment at all time points measured (n = 6 rats/group). *P ≤ 0.05, **P ≤ 0.01. Blue Filled circle—vehicle, red filled triangle—fostamatinib.

Following establishment of a sensitized model, the role of fostamatinib in DSA production was investigated. Sensitized rats were treated with fostamatinib 24 h after blood transfusion. Flow crossmatch analysis demonstrated that SYK inhibitor treatment was able to block production of significant levels of IgM (Fig. 2A), IgG (Fig. 2B) and IgG subsets IgG1 (Fig. 2C), IgG2a (Fig. 2D), IgG2b (Fig. 2E) and IgG2c (Fig. 2F) throughout the course of treatment.

### Total circulating IgG and IgM levels are not affected by fostamatinib

**Figure 3:**
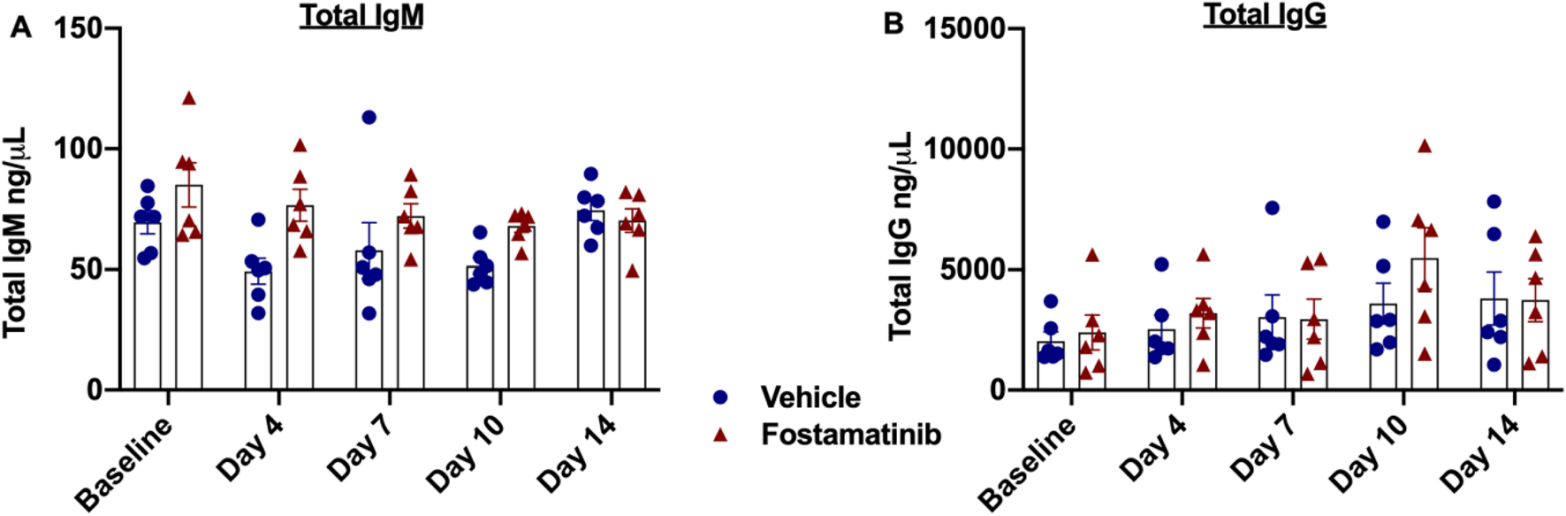
Fostamatinib treatment had no effect on total circulating IgM and IgG. Serum was analysed by ELISA for (**A**) total IgM and (**B**) total IgG levels. There was no significant difference at any measured time point between fostamatinib treated rats and vehicle treated rats for both IgM and IgG levels (n = 6 rats/group). Blue filled circle—vehicle, red filled triangle—fostamatinib.

As fostamatinib prevented the formation of an alloantibody response, serum was analysed for total circulating IgM (Fig. 3A) and IgG levels (Fig. 3B). These levels remained comparable between treatment groups.

### Fostamatinib treatment did not affect mature B lymphocyte subsets or plasma cell numbers

**Figure 4:**
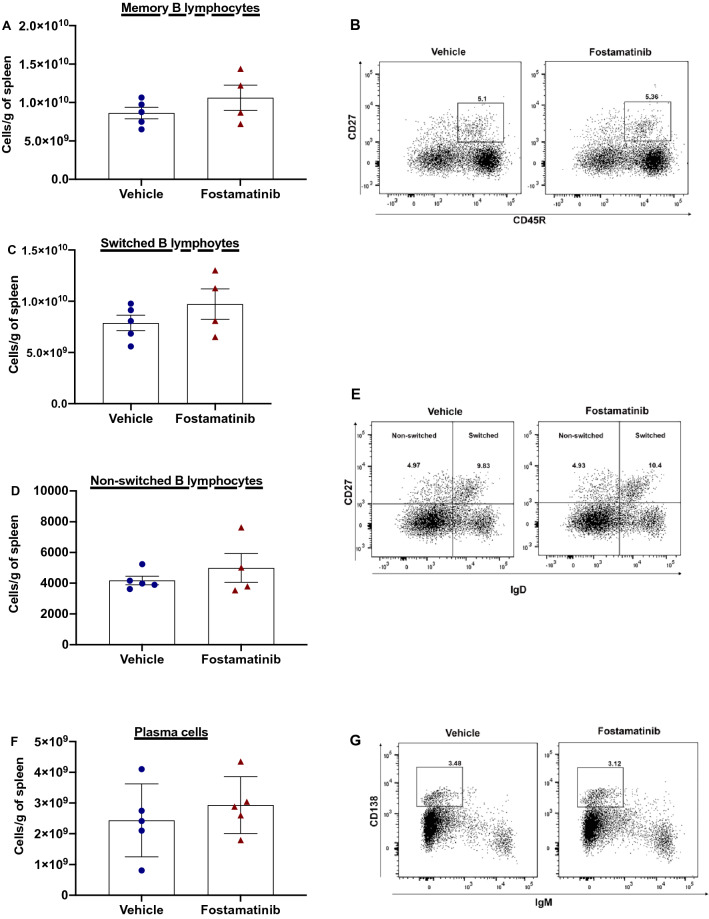
Fostamatinib treatment did not affect B cell or plasma cell populations. Flow cytometry was used to measure mature B lymphocytes populations in fostamatinib treated and vehicle treated sensitized LEW rats. Each graph shows number of cells per gram of spleen. Mature B lymphocyte numbers remained similar between treatment groups. (**A**) Memory cells were defined as CD45R^+^CD27^+^, (**C**) switched cells as CD45R^+^CD27^+^IgD^−^, (**D**) non-switched cells CD45R^−^CD27^+^ IgD^+^, (**F**) plasma cells as IgD^−^CD45R^−^IgM^−^CD138^+^. (n = 6 rats/group). Graphs show cell numbers per gram of spleen (n = 6 rats/group). Representative flow cytometry data of (**B**) memory B lymphocytes (**E**) switched and non-switched B lymphocytes (**G**) plasma cells. Number shown represents percentage of cells in gate. Blue filled circle—vehicle, red filled triangle—fostamatinib.

Splenic mature B lymphocyte populations were evaluated. Memory B lymphocytes (CD45R^+^CD27^+^) (Fig. 4A,B), switched cells (CD45R^+^CD27^+^IgD^−^) (Fig. 4C,E) and non-switched cells (CD45R^+^CD27^+^IgD^+^) (Fig. 4D,E), were detected at similar levels in both treatment groups compared to control rats. Given that DSA production was profoundly inhibited by fostamatinib, effects on numbers of splenic plasma cells were evaluated between treatment groups and compared to control non-sensitized LEW rats (Fig. 4F,G). Unlike DSA production, plasma cell numbers (CD138^+^CD45R^−^) were not affected by fostamatinib therapy and both treatment groups remained at similar levels compared to non-treated non-sensitized animals.

### Fostamatinib prevented DSA levels rising 5 weeks after termination of treatment

**Figure 5:**
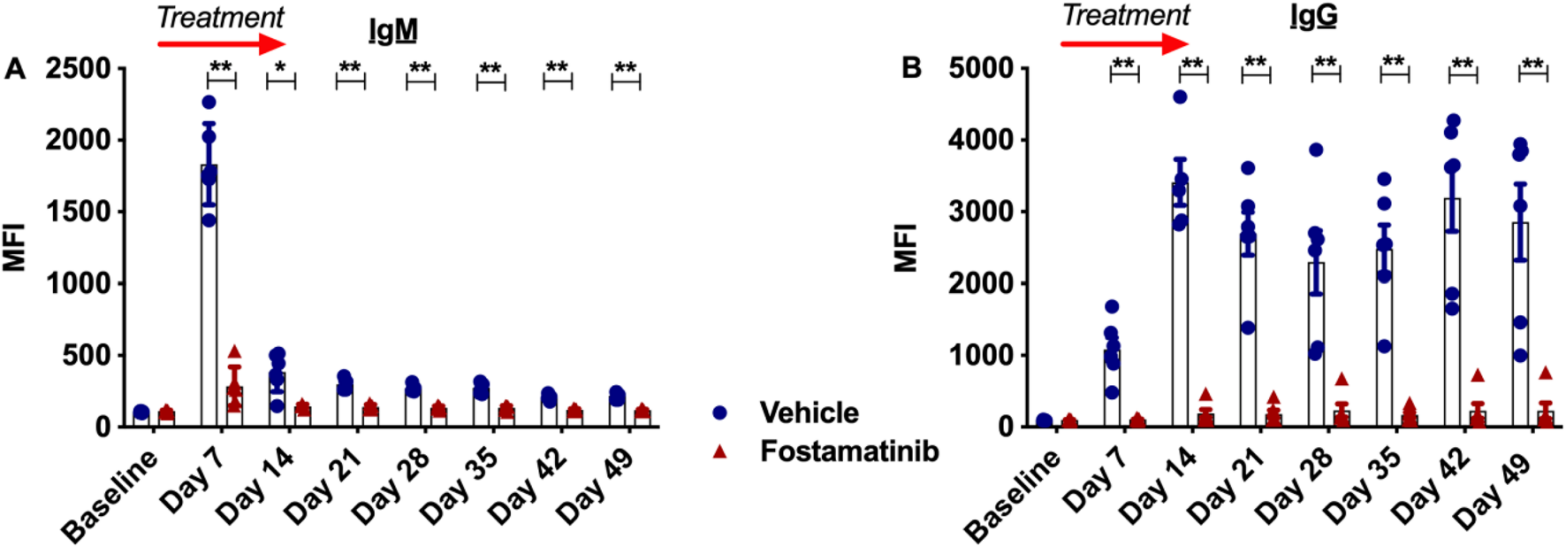
Fostamatinib prevents DSA production up to 5 weeks post completion of treatment. Treatment was given from day 1 to day 14 after blood transfusion. Then, the antibody levels were monitored until day 49. T lymphocyte crossmatch analysis was performed to detect DSA levels in sensitized fostamatinib and vehicle dosed LEW rats. (**A**) IgM and (**B**) IgG production was inhibited up to 5 weeks post-termination of the fostamatinib treatment regimen (n = 6 rats/group) (*P ≤ 0.05, **P ≤ 0.01). Blue filled circle—vehicle, red filled triangle—fostamatinib.

Following potent inhibition of DSA levels with fostamatinib treatment, we wanted to evaluate if this suppression was transient or maintained. Sensitized rats were treated with fostamatinib for the 2-week regimen as described previously and, upon completion of the treatment course, rats were left untreated for 5 weeks with weekly venesection for measurement of DSA levels. T lymphocyte crossmatch analysis of serum showed that 2-week fostamatinib treatment was able to maintain suppression of formation of DSA IgM (Fig. 5A) and IgG (Fig. 5B) up to 5 weeks after completion of the treatment regimen.

### Delayed fostamatinib treatment prevented IgG DSA reaching levels found in vehicle treated rats

**Figure 6:**
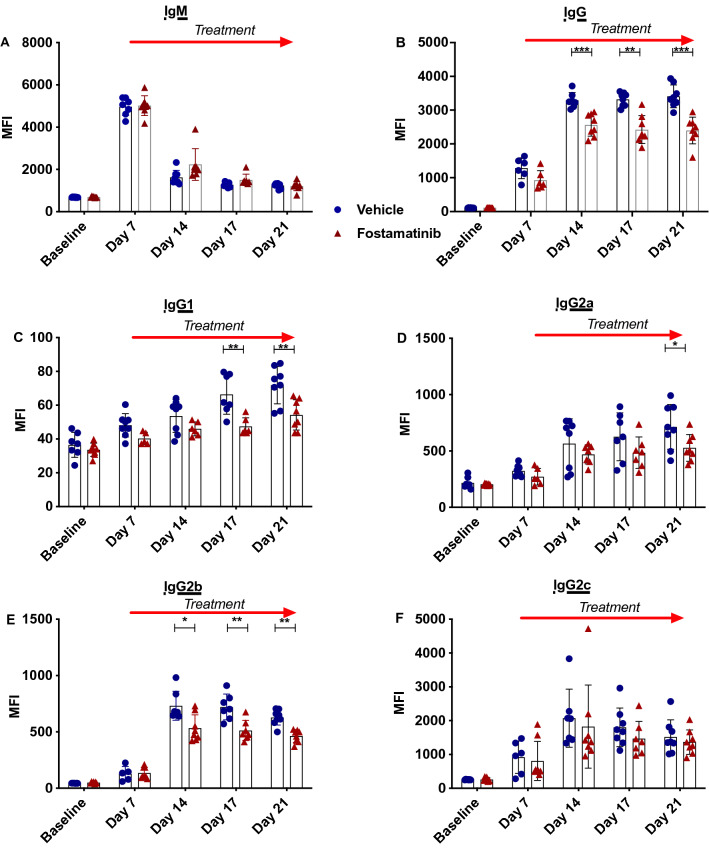
Delayed fostamatinib treatment reduces levels of DSA IgG in sensitised rats. T lymphocyte crossmatch analysis was performed to detect DSA levels in sensitized fostamatinib and vehicle treated LEW rats. Treatment initiation was delayed until 7 days post-transfusion. (**A**) IgM levels remained the same in both treatment groups. (**B**) Circulating allogenic IgG levels were significantly lower in fostamatinib treated rats at days 14, 17 and 21 post-transfusion. (**C**) From 17 days onwards IgG1 levels were significantly lower in fostamatinib treated animals, from 14 days onwards IgG2a (**D**) levels were significantly lower in fostamatinib treated animals, and by 21 days post-transfusion IgG2b (**E**) levels were significantly lower in fostamatinib treated animals. No differences were observed in IgG2c levels at any measured time point (**F**) (n = 6 rats/group) *P ≤ 0.05, **P ≤ 0.01, ***P ≤ 0.001). Blue filled circle—vehicle, red filled triangle—fostamatinib.

To assess the efficacy of fostamatinib treatment initiated at a later timepoint in sensitized rats, the fostamatinib treatment regimen was delayed until 7 days post-transfusion. Results showed that fostamatinib did not block allogenic IgM production and circulating levels were comparable between treatment groups (Fig. 6A). However allogenic IgG levels were significantly reduced from day 14 in fostamatinib treated rats (Fig. 6B). Investigating this further, IgG subsets were measured, demonstrating variable results (Fig. 6C–F). Levels of IgG2b, IgG1 and IgG2a showed significant blocking of alloantibody production from days 14, 17 and 21 respectively. IgG2c showed no difference between treatment groups at any time point.

## Discussion

In this study, we demonstrate for the first time that SYK inhibition with fostamatinib significantly prevents allogenic DSA production in a rat model of sensitization. Fostamatinib treatment initiated 24 h post-transfusion for the treatment regimen of 2 weeks was effective at preventing production of both allogenic IgG and IgM, and had no depletory effects on total IgG and IgM levels. Fostamatinib treatment remained effective even 5 weeks post-termination of treatment, with significantly lower levels of IgG and IgM detected by the T cell crossmatch test at this time point. Fostamatinib was also able to partially block DSA production when treatment was implemented 7 days post-sensitisation, after the initiation of an allogenic antibody response. Our study shows that fostamatinib had a direct effect on inhibition of alloantibody without adversely affecting B cell survival. Splenic plasma cell numbers were comparable between both treatment groups. In addition to plasma cells, we examined the effect of SYK blockade on other mature B lymphocyte populations. Memory, switched and non-switched populations of B cells in the spleen remained similar between both treatment groups. Normal B cell activation and development is therefore not being affected by fostamatinib treatment, rather the production of allogenic antibody is.

SYK has a well-defined role in signal transduction downstream of immunoreceptors and is critical in mediating BCR responses and FCR responses in mast cells^20^, dendritic cells^20^, macrophages^21^ and neutrophils^22^. The SYK homologue zeta chain-associated protein kinase 70 (ZAP-70)^23^ is the predominant signalling molecule downstream of the TCR in T cells and natural killer cells. Studies involving SYK knockout murine models have shown the critical role of SYK signalling for B cell development and maturation, where B cell maturation is arrested from progressing at the early pro-B-cell state in murine SYK knockout models^11, 24, 25^. Early mechanistic data has demonstrated that, in vitro fostamatinib was able to inhibit BCR responses in primary human B cells, where CD69 cell surface upregulation induced by BCR crosslinking with IgM was inhibited with fostamatinib treatment^26^.

The role of SYK in antibody production in plasma cells is unclear, as B cells from SYK knockout mice are unable to mature beyond the pro-B-cell stage^25^. In vivo models have provided some insight and indications that SYK inhibition may be an effective target for the treatment of antibody-mediated diseases. In a rodent model of anti-glomerular basement membrane disease, R406 treatment prevented the induction of disease, and inhibited circulating and deposited autoantibody production in established disease^19^. Interestingly in this model, CD45RA^+^ cell numbers remained the same despite decrease of autoantibody. Non-obese diabetic mice were protected against developing diabetes with fostamatinib treatment in a prevention setting. Treatment was also able to delay disease progression in glucose intolerant mice, with a measured reduction in anti-glutamic-acid-decarboxylase anti-islet antibodies^27^.

Currently there are no reported data on the efficacy of fostamatinib in reduction of alloantibody production, and to our knowledge this is the first report of its kind. In contrast to our experiments, early immunotoxicology studies where rats were immunised with KLH-Ribi antigen and treated with fostamatinib, did not show reduced KLH specific IgM and IgG^28^. Differences between the effects of fostamatinib on production of alloantibody compared to autoantibody are intriguing and require further investigation. The mechanism of inhibition of DSA production by fostamatinib in our experiments is unclear. Due to the functional action of fostamatinib, it is likely that downstream signalling molecules have been blocked from phosphorylation, which in-turn has impacted DSA production. Fostamatinib inhibition of SYK prevents phosphorylation of downstream molecules, which include phospholipase Cγ1, Akt/protein kinase B, c-Jun N-terminal kinase, p38 and extracellular signal-regulated kinase^26^. Exactly which signals contribute to immunoglobulin production requires further investigation. It is possible in our experiments that fostamatinib treatment is blocking antigen presentation to B cells via follicular dendritic cells, a process required for the progression of the B cell response. Studies have also established a crucial role for SYK signalling in the induction of the antigen presentation machinery in both B cells^27^ and dendritic cells^29^. In dendritic cells, SYK signalling has shown to be crucial for immunocomplex uptake and antigen presentation^20^.

In our model, blood transfusion initially induced IgM production, with peak titres approximately 7 days post sensitization. Traditionally in the context of AMR, preformed IgM antibodies have been perceived to be non-pathogenic. However emerging studies suggest that anti-HLA IgM may make important contributions to the pathogenesis of allograft loss^30–33^. In our experiments where treatment was implemented 24 h post sensitization, DSA IgM production was blocked, indicating this might be beneficial for human presensitization. Fostamatinib did not block IgM levels when treatment was initiated at a later time-point, as class switching had converted production to the IgG class.

Rodents have four IgG subclasses IgG1, IgG2a, IgG2b and IgG2c^34^. IgG2a and IgG2b have been reported to have the strongest complement fixing ability^35^. In humans four IgG subclasses exist, IgG1, IgG2, IgG3 and IgG4. It is widely accepted that IgG1 and IgG3 are the most pathogenic subclasses in AMR due to their ability to activate the complement cascade^36^. IgG_3_ has the highest binding efficiency to complement component C1q, and IgG1 is highly effective at complement dependent cell lysis^37^. The presence of these subclasses as a result of sensitization is associated with poor graft outcome^33^. In early treatment experiments we have shown the efficacy of fostamatinib in blocking production of all IgG subtypes, thereby resulting in prevention of downstream effects of humoral immunity, suggesting this approach could be suitable in prevention of presensitization when given concomitantly to blood transfusions or possibly de novo DSA during transplantation^38^. In addition to DSA level reduction, utilizing fostamatinib to inhibit the effector phase and ultimately influencing the pathogenicity of the DSA underpins the rationale for using fostamatinib in AMR.

Fostamatinib treatment initiated 7 days post transfusion was able to significantly reduce the levels of DSA IgG detected in the CD3^+^ crossmatch assay at days 14, 17 and 21. Study of IgG subsets provided variable results. IgG2c levels were similar between groups, but IgG1 and the most pathogenic subsets IgG2a and IgG2b demonstrated significantly lower levels in treated rats from at day 14. It is probable that fostamatinib treatment was inhibitory soon after administration, as demonstrated by early time point experiments, however fostamatinib is unlikely to inhibit DSA IgG that has already been made. The circulating half-life of IgG is 7–25 days and it is likely that an extended follow-up time would continue to show more pronounced repression of DSA IgG levels. The ability of fostamatinib to block de novo DSA IgG production but not existing DSA IgG could explain the substantially higher DSA IgG levels measured in late treatment experiments compared to earlier treatment experiments.

In the context of blood transfusion as a sensitizing event, there is an abundance of literature associating allosensitization with higher rates of graft rejection and lower rates of graft survival^39^. Chronic anaemia is prevalent in patients with chronic kidney disease or end-stage kidney disease and avoiding transfusion is not always feasible^40^. A causal link between blood transfusions and DSA production has been identified by Hassan et al.^41^. In patients receiving blood transfusion after renal allograft transplantation, this study found direct evidence of a de novo HLA alloimmune response elicited against the blood donor, and enhanced risk of transplant specific antibody development within these patients. These factors were significantly associated with the increased risk of AMR and allograft failure. Current treatment options including early post-transplant erythropoietin therapy and cell salvage have limited efficacy^42^.

In a pre-transplant setting, current treatment strategies for sensitized patients have partial or transient efficacy and include the use of plasma exchange, anti-CD20 monoclonal antibody therapy with rituximab, or blocking macrophage FcγR with high-dose intravenous immunoglobulin^43^. Fostamatinib treatment presents a potential alternative strategy in preventing allosensitization in both pre-transplant and post-transplant blood transfusions. In our experiments, a relatively transient period of orally given treatment was able to inhibit DSA levels even up to 5 weeks post sensitization, which is significant in a clinical context as it is likely to improve patient compliance compared to more regular or invasive treatment. Our data are limited by the moderately short follow up time, and the long-term impact of SYK inhibition on plasma cell and B cell function is unknown. Further work will be needed to investigate whether treatment with fostamatinib may have a role in treatment of sensitized rats in models of experimental transplant rejection.

LEW and F344 rats are weakly histocompatible due to differing partially at both MHC I & II and non-MHC loci^44^. Transplantation of an F344 kidney into a LEW rat is a well-characterized model of chronic antibody mediated rejection, where rejection develops over the course of a few months^45^. It will be crucial to investigate the protective potential of fostamatinib in sensitized transplanted rats from developing chronic AMR. In a rat model of acute rejection, involving the Dark Agouti (DA/Arc, RT1^av1^) kidney transplanted into the LEW, SYK inhibition demonstrated the ability to reduce acute injury in transplanted renal allografts in sensitized recipients^46^. This study explored the efficacy of SYK inhibitor GS-492429 in acute rejection, with a splenocyte transfusion for the sensitization method. The protective results are promising for our future studies in a chronic AMR rat model. Fostamatinib is currently FDA approved for the treatment of thrombocytopenia in adults with chronic ITP with insufficient response to other treatment. Appropriate dosing, PKA data and a good safety profile for fostamatinib are available^47^. The data presented in this study suggest that fostamatinib should be investigated for reduction of circulating allogenic antibodies in patients with chronic kidney disease needing blood transfusion and highly sensitized patients with end-stage renal disease awaiting transplant.

## Data Availability

The data supporting the findings of this study is available from the corresponding author upon reasonable request.
